# Rethinking *Candida* Vaccine Translation: Barriers, Immunotherapies and a One Health Imperative

**DOI:** 10.3390/vaccines14090825

**Published:** 2026-09-19

**Authors:** Julie Krier, Francisco A. M. Silva, Célia Fortuna Rodrigues

**Affiliations:** 1 Department of Pharmaceutical Sciences, CESPU University, 4585-116 Gandra, Portugal; a31862@alunos.cespu.pt (J.K.); francisco.silva@iucs.cespu.pt (F.A.M.S.); 2 Associate Laboratory i4HB—Institute for Health and Bioeconomy, CESPU University, 4585-116 Gandra, Portugal; 3 UCIBIO—Research Unit on Applied Molecular Biosciences Unit, Translational Toxicology Research Laboratory, CESPU University, 4585-116 Gandra, Portugal; 4 LEPABE/ALiCE—Laboratory for Process Engineering, Environment, Biotechnology and Energy and Associate Laboratory in Chemical Engineering, Faculty of Engineering, University of Porto, Rua Dr. Roberto Frias, 4200-465 Porto, Portugal

**Keywords:** *Candida*, vaccine, *Candida auris*, antifungal resistance, translational barriers, immunotherapy, One Health

## Abstract

Over the past decade, antifungal vaccinology has produced genuine proof of concept: adhesin-based candidates such as NDV-3A have completed Phase II human trials, and reverse-vaccinology pipelines now routinely generate multi-epitope constructs with high predicted population coverage against *Candida albicans* and the multidrug-resistant *Candida auris*. However, no antifungal vaccine has reached the market, and the reasons for this gap are rarely examined together in a single, critical account. This review argues that the bottleneck is not primarily antigen discovery, but a layered set of translational barriers: biological constraints inherent to a commensal, morphologically plastic pathogen; manufacturing and purity requirements that complicate subunit vaccine production; a historical bias in preclinical animal models toward chemically immunosuppressed, rather than genetically susceptible, hosts; an underutilization of key insights from veterinary medicine; and a fragmented regulatory and commercial landscape that structurally disincentivizes investment in niche, high-risk patient populations. We further examine emerging immunotherapeutic strategies—passive antibody engineering, cytokine-based adjuvant approaches, phytocompound-derived antifungals, and dual-action nanovaccines—as partial, rather than complete, solutions to the immunocompromised-host paradox that limits active immunization in the patients who need protection most. Finally, we situate *Candida* vaccine development within a One Health and global-equity framework, arguing that diagnostic infrastructure, HLA population diversity, and cold-chain economics must be addressed as part of vaccine design rather than as an afterthought.

## 1. Introduction

Invasive candidiasis remains one of the deadliest and most persistent healthcare-associated infections, with an estimated 250,000 to 700,000 cases occurring annually worldwide and mortality rates ranging from 40% to 55% [1,2]. In developed healthcare systems, it continues to rank among the most common hospital-acquired fungal infections, and its epidemiological profile has shifted substantially over the past decade: *Candida albicans* is gradually being displaced by intrinsically resistant non-*Candida albicans Candida* (NCAC) species, most notably *Candida glabrata* and the multidrug-resistant *Candida auris* [1,3,4]. Against this backdrop, prophylactic vaccination has long been proposed as the most durable solution—one capable of protecting patients before immunosuppression begins, rather than treating infection after the fact.

Substantial scientific progress supports this vision. The NDV-3A vaccine, built around the recombinant N-terminus of the Als3 adhesin, has completed a randomized, placebo-controlled Phase II trial and demonstrated cross-protection against *C. auris* in preclinical models [5,6]. Reverse-vaccinology platforms now generate multi-epitope candidates engineered for broad HLA population coverage entirely *in silico*, bypassing the need to culture highly pathogenic strains [4,7,8,9]. These advances are the subject of a growing and increasingly detailed literature on antigen selection and delivery-platform design.

The objective of this review is threefold, linked by a direct causal sequence. First, layered biological, regulatory, and manufacturing barriers frequently render conventional active prophylactic vaccines ineffective in deeply immunocompromised target populations. Second, alternative immunotherapies, such as passive monoclonal antibodies, cytokine adjuvants, and nanovaccines, offer critical workarounds to bypass host immune paralysis, yet remain constrained by high biomanufacturing costs and clinical delivery limits. Consequently, embedding One Health equity constraints, such as cold-chain stability, broad HLA population coverage, and diagnostic infrastructure, early into initial vaccine R&D is essential to ensure that emerging candidates remain viable and globally accessible across resource-limited settings [10,11,12,13,14,15].

While recent reviews (2020–2025) have extensively covered antigen discovery and delivery platforms [4,7,16], they rarely analyze why these candidates consistently stall before clinical implementation. Existing literature typically addresses individual obstacles, such as financial risk or preclinical model selection, in isolation. This review fills this gap by proposing a unified, three-tiered framework. By systematically connecting structural and manufacturing barriers, alternative immunotherapeutic workarounds, and One Health equity constraints within a single critical analysis, we provide a systems-level perspective on what is required to translate *Candida* vaccine candidates into accessible clinical tools.

## 2. Methods

For this narrative review, peer-reviewed literature published in English between 2015 and 2025 was retrieved from PubMed and ScienceDirect. This window was chosen to capture the period following the first Phase 2 trial of NDV-3A (2018) and the subsequent emergence of *Candida auris* as a global public health concern. Search terms combined “Candida” or “Candida auris” with terms specific to each of the three thematic pillars addressed here: for structural and clinical barriers, “translational barriers,” “antifungal resistance,” “regulatory,” and “clinical trial design”; for emerging immunotherapeutic strategies, “passive immunization,” “monoclonal antibody,” “dendritic cell,” “cytokine adjuvant,” “nanovaccine,” and “antifungal agent”; and for the One Health dimension, “One Health,” “diagnostics,” “HLA,” and “global health equity”—each combined with “fungal” or “antifungal.” To ensure reproducibility, these terms were operationalized as explicit Boolean search strings, adapted to each database’s syntax. In PubMed/MEDLINE: ((“Candida”[MeSH Terms] OR “Candida auris”) AND (“vaccine”[MeSH Terms] OR “immunization” OR “immunotherapy”)) AND (“translational barriers” OR “antifungal resistance” OR “regulatory” OR “clinical trial design” OR “passive immunization” OR “monoclonal antibody” OR “dendritic cell” OR “cytokine adjuvant” OR “nanovaccine” OR “One Health” OR “diagnostics” OR “HLA” OR “global health equity”) AND (“fungal” OR “antifungal”)

**Figure 1:**
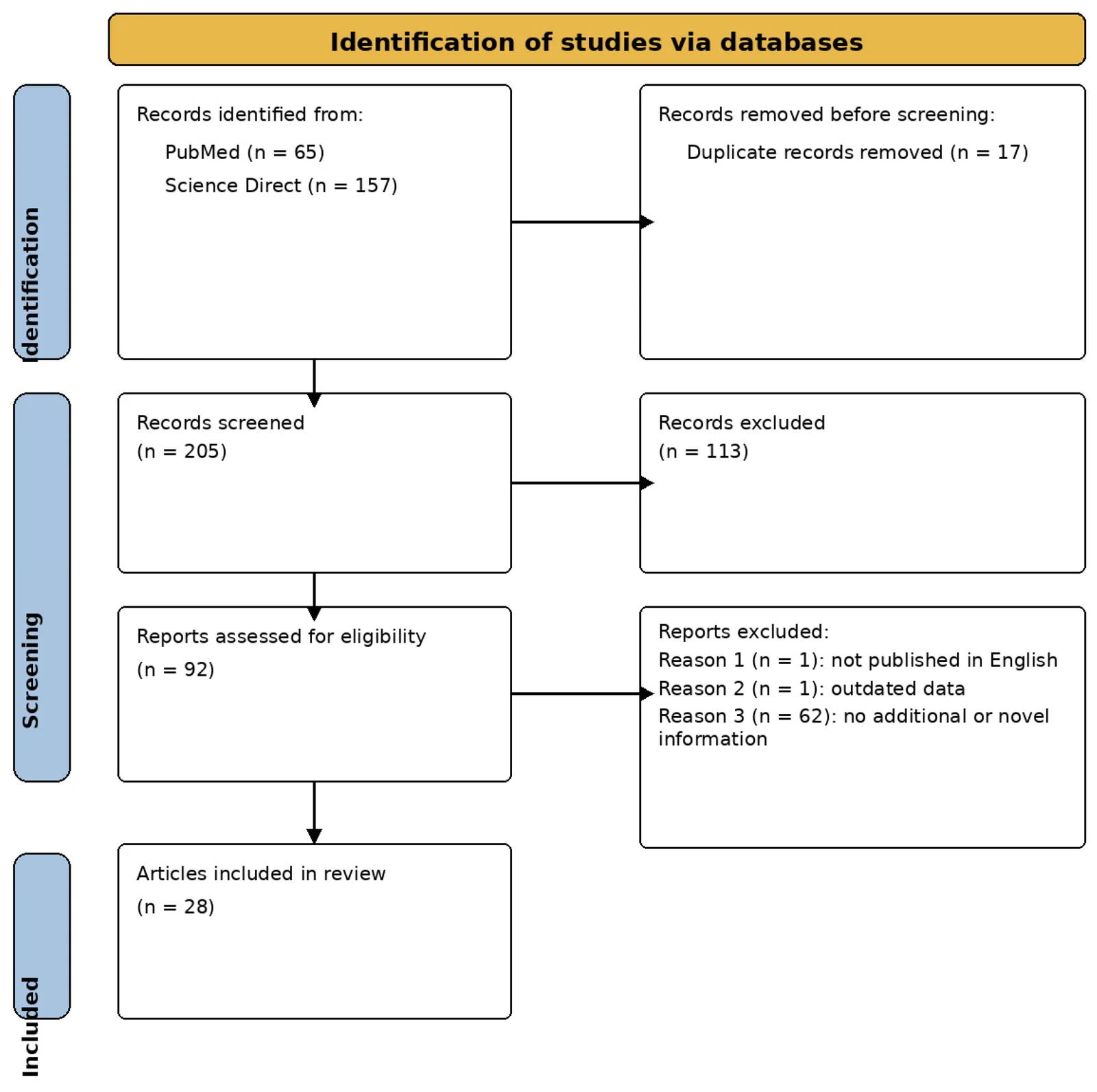
PRISMA flow diagram of the study selection process.

An analogous string, adapted to the corresponding indexing and title/abstract search conventions of ScienceDirect, was used in that database. Articles were included if they reported primary experimental, clinical, or translational data, or provided directly relevant expert analysis, on any of the three thematic pillars; non-English-language publications, conference abstracts without an accompanying full text, and non-peer-reviewed sources were excluded. The study selection process is summarized in Figure 1.

## 3. Structural and Clinical Barriers to Translation in *Candida* Vaccines

The global clinical landscape of fungal infections is changing rapidly, with marked disparities between high-income countries (HICs) and low- and middle-income countries (LMICs). In LMICs, invasive candidiasis exhibits significantly higher crude mortality rates (reaching 40% to 60%) compared to HICs, driven by limited access to rapid diagnostic tools and costly first-line echinocandins [1,2]. Epidemiologically, *C. albicans* is gradually being displaced by non-*Candida albicans Candida* (NCAC) species: fluconazole-resistant *C. parapsilosis*, and *C. tropicalis* dominate LMIC clinical settings, whereas multidrug-resistant *C. auris* has emerged globally with distinct regional clade dynamics, triggering severe, persistent hospital outbreaks in resource-constrained intensive care units [1,3,4]. This shifting, region-specific epidemiological profile (Table 1) underscores the need for vaccines that offer broad cross-protection and remain viable across diverse healthcare infrastructures.

Therefore, widespread, long-term use of traditional antifungal drugs has led to an alarming rise in resistance. This growing loss of drug sensitivity highlights the urgent need for immunobiological alternatives like vaccines [10]. Recent clinical data show that the emergence of multidrug-resistant NCACs has severely reduced the efficacy of first-line treatments. Consequently, hospital mortality rates for systemic infections still oscillate between 30% and 50% [11]. Vaccines and related immunobiologicals should therefore be regarded as potential preventive or adjunct immunotherapeutic strategies for settings where conventional drugs are failing, rather than as established therapeutic options.

A critical barrier to safety is the risk of vaccine-induced autoimmunity driven by molecular mimicry. Fungal target antigens often share structural homology with human proteins. To prevent cross-reactive autoimmune attacks against the host proteome, current antigen discovery pipelines rely on strict bioinformatic filtering. This processing isolates highly specific fungal epitopes while systematically discarding sequences that resemble human proteins [7]. This autoimmune risk is particularly high when targeting evolutionarily conserved proteins, such as Hsp90 [12]. Because Hsp90 acts as an essential molecular chaperone in both eukaryotic fungi, inducing a powerful antibody or T-cell response against it carries an intrinsic hazard of auto-reactivity.

To safely exploit this highly immunogenic target, investigators must perform exhaustive epitope mapping. Advanced bioinformatics are mandatory to isolate strictly fungus-specific protein domains before moving into human clinical trials [12,13].

Finally, manufacturing purity dictates the overall safety profile of the final biopharmaceutical product [14,15]. The presence of manufacturing remnants, known as “ballast” substances, which include cell wall debris, host cell proteins, and culture impurities, can trigger severe, non-specific adverse reactions *in vivo.* For this reason, subunit vaccines are highly preferred over crude cell homogenates [16,17,18,19,20,21,22,23,24,25,26]. Utilizing precise mechanical cell disruption followed by multi-stage ultrafiltration techniques effectively minimizes these unwanted contaminants, ensuring a clean, highly predictable, and safe clinical profile [10].

Importantly, the discussion regarding human antifungal vaccine development can be significantly enriched by considering the extensive experience of veterinary medicine in fungal immunoprophylaxis. Veterinary medicine has provided practical proof-of-concept for livestock fungal vaccines (most notably against dermatophytosis such as *Tricophyton verrucosum*), demonstrating that targeted fungal immunization is technically feasible at scale [16,17,18,19,27,28]. This field highlights the critical importance of rigorous antigen standardization, preclinical evaluation of immunogenicity and allergenicity, and the strategic use of adjuvants to elicit effective cell-mediated immunity [28].

However, analyzing why similar anti-*Candida* developments or broader systemic fungal vaccines have not been widely adopted in veterinary medicine sheds light on potential barriers to scaling up these technologies. In the veterinary setting, candidiasis is primarily an opportunistic co-infection rather than an epidemic herd disease, making the high production costs of standardized eukaryotic antigens financially unviable compared to conventional chemical antifungals. Furthermore, challenges related to antigen batch-to-batch stability, complex scaling of sub-cellular components, and variable efficacy in immunocompromised animals closely parallel the translational bottlenecks currently observed in human clinical trial development [24,26,28].

A persistent technical barrier in rodent testing has been the historic absence of animal models that mimic *C. auris* infection without using chemical immunosuppression. Most standard models of disseminated candidiasis traditionally used C57BL/6J mice. Because this strain is naturally resistant to *C. auris*, researchers had to inject high doses of cyclophosphamide to induce pathology (Table 2). This practice creates an experimental bias. Severe chemical immunosuppression completely prevents an accurate assessment of how a vaccine candidate interacts with a functional, intact immune network.

The recent characterization of the A/J mouse model, which is naturally deficient in the C5 complement component, has finally addressed this gap [13]. This model demonstrated that host resistance to this emerging pathogen depends critically on the complement cascade. Discovering that A/J mice succumb to *C. auris* in a fulminant manner, while other specialized models (such as those deficient in neutrophil elastase) remain resistant, reveals that the choice of animal strain has been an underestimated barrier in the past [13].

Without animal models that leverage the natural virulence of the fungus in hosts with precise, targeted genetic deficiencies rather than total chemical destruction, transitioning vaccine candidates to human clinical trials will continue to face high failure rates due to inaccurate preclinical predictions.

The transition of antifungal vaccine candidates from laboratory research to large-scale clinical success is heavily hindered by deep logistical, diagnostic, and regulatory barriers (Table 3). While preclinical pipelines generate promising candidates, the scarcity of human clinical data remains a primary bottleneck [14]. This challenge is made worse by a severe diagnostic limitation in current medical mycology: the low sensitivity of traditional blood cultures.

Blood cultures catch only about 50% of active invasive candidiasis cases [1]. This endemic diagnostic gap introduces a critical delay of 24 to 72 h before clinicians receive a confirmatory laboratory result. This window not only prevents early therapeutic intervention but also obscures the precise clinical endpoints required to accurately evaluate the protective success of new immunobiological tools during large-scale trials [11]. Because classical microbiology is too slow, clinical research is exploring new risk-stratification tools based on host genetic screening. Identifying specific SNPs linked to fungal susceptibility has been proposed as a candidate predictive biomarker network [15]. Integrating rapid genetic screening could, in principle, help clinicians identify high-risk patients before the onset of physical symptoms or blood-culture positivity, but this remains a potential future approach for risk stratification that still requires prospective clinical validation before it could mitigate the impact of diagnostic delays on patient selection during trials.

This diagnostic barrier is further exacerbated by an uneven distribution of laboratory resources. A widespread lack of specialized reagents, automated identification equipment, and mycological expertise in many healthcare facilities prevents the accurate identification of the specific fungi driving an infection. Consequently, deep candidiasis is frequently diagnosed as a generic fungal infection. This lack of species-level resolution completely hinders the deployment of advanced, species-specific vaccines, which require precise microbial identification to work (Table 4) [10].

Furthermore, the inherent complexity of designing clinical trials for antifungal vaccines creates a massive regulatory hurdle. Unlike universal prophylactic viral vaccines, *Candida* candidates target highly fragmented and heterogeneous patient cohorts, such as distinct groups of immunosuppressed individuals. This fragmentation makes standardizing international clinical protocols and securing approval from regulatory bodies, like the FDA or EMA, exceptionally complicated [12]. Finally, the transition to clinical practice is slowed by the biological and physical properties of advanced antigen delivery systems, such as nanoparticles and engineered hydrogels. These modern platforms have complex *in vivo* pharmacokinetics and often suffer from formulation instability during storage [14].

**Figure 2:**
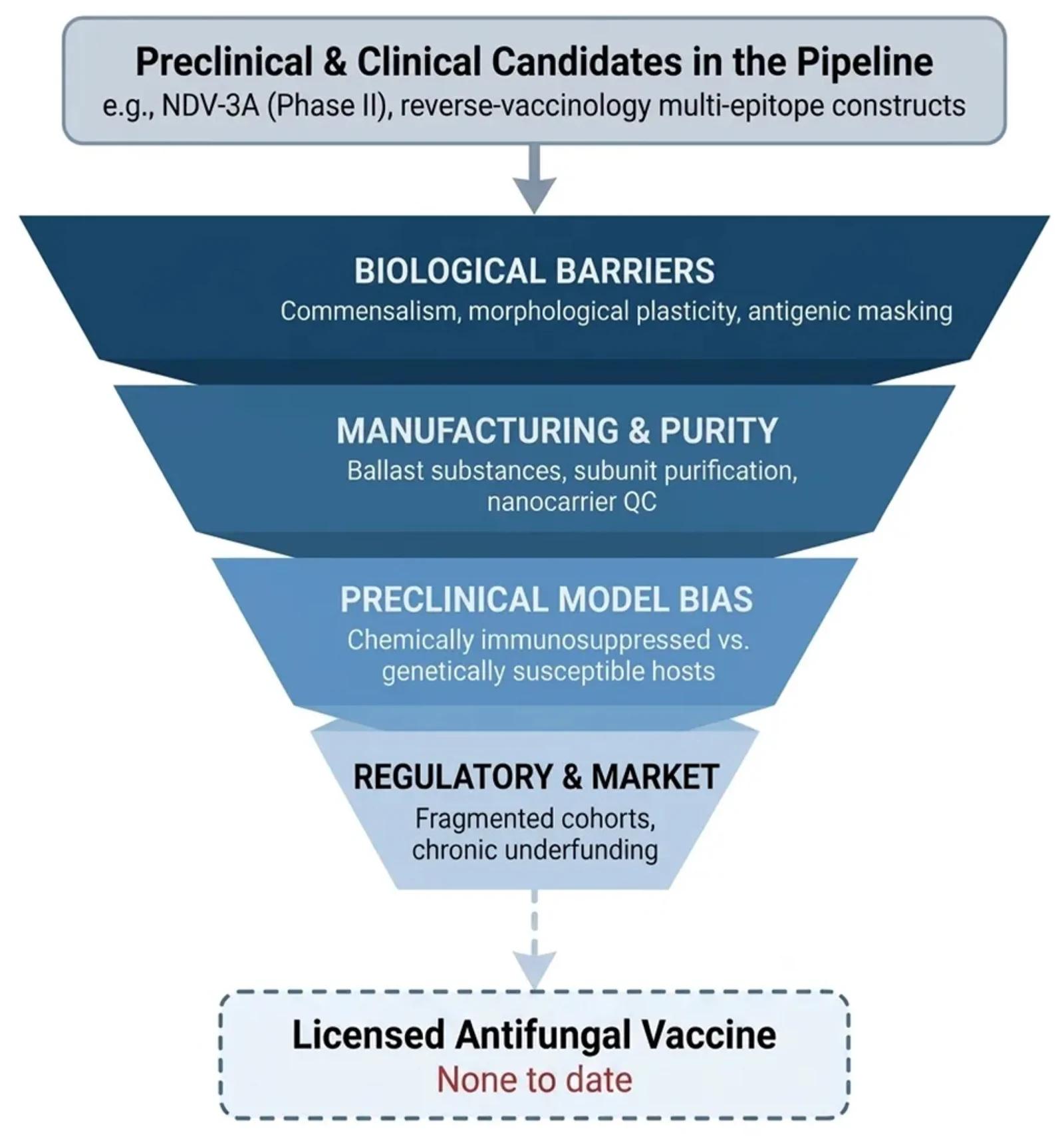
From pipeline candidates to licensed product: the four layered barriers narrow the funnel from promising preclinical/clinical candidates toward a licensed antifungal *Candida* vaccine.

Because these delivery materials still lack long-term, large-scale human safety profiles, regulatory agencies require exhaustive toxicity data. This requirement creates prolonged approval timelines that slow down industrial investment and clinical implementation (Figure 2) [14].

Although advanced candidates like NDV-3A show a promising capacity to cross-protect against *C. auris*, vaccines designed from the ground up to target these emerging species remain rare [14].

From a macroeconomic perspective, a critical gap exists between academic discovery and industrial manufacturing. The pharmaceutical sector consistently underinvests in fungal vaccines compared to viral or bacterial platforms, effectively blocking promising candidates from entering final-phase human testing [4]. This industrial reluctance stems primarily from the massive financial costs associated with the expression, purification, and quality control of complex eukaryotic macromolecules. This high upfront investment is paired with the perception of an uncertain, low market return on investment.

Because the primary market for a *Candida* vaccine is restricted to specific, fragmented patient niches, such as ICU cohorts, transplant recipients, or individuals undergoing chemotherapy, rather than the healthy global public, large pharmaceutical companies systematically prioritize funding broad-spectrum viral or bacterial programs. This financial bias perpetuates a state of chronic underfunding. It slows down the transition of top-tier academic vaccine candidates into real-world clinical availability.

The clinical landscape has expanded by exploring diverse technological platforms beyond traditional recombinant proteins (Table 5). Current pipelines include the use of glucan particles and highly specific fungal cell wall glycoproteins to improve antigen presentation [16]. Modern vaccine engineering emphasizes the importance of driving a balanced T-cell response through the intentional activation of host dendritic cells. This reflects a paradigm shift from simple antibody production toward establishing a more complex, long-lasting immunological memory [16]. These new clinical designs also focus on maximizing the biophysical profiles of the candidates. Optimizing parameters such as protein hydrophilicity and long-term thermostability remains essential to ensure that future formulations can endure large-scale industrial manufacturing.

Despite historical milestones, the definitive transition to clinical practice faces persistent challenges. The primary obstacle remains translating results from controlled animal models to deeply immunocompromised human patients. Most current clinical successes, including NDV-3A, were validated in immunocompetent or partially immunocompetent individuals, such as women with mucosal RVVC [4,16].

Demonstrating vaccine efficacy in profoundly neutropenic populations remains a major barrier. In these patients, the severe shortage or complete absence of innate effector cells severely limits the impact of vaccine-induced antibodies, which require functional white blood cells to achieve pathogen clearance [16]. The Phase II clinical trial of NDV-3A highlights the difficulty of defining and achieving clear efficacy endpoints in humans [6]. The study demonstrated that intrinsic demographic factors, such as being over the age of 40, can significantly mask the overall statistical efficacy of an immunogen. This age-dependent variation creates additional hurdles when designing future clinical protocols. It requires a much more refined selection of patient cohorts during phase enrollment to avoid confounding variables linked to natural immune senescence. Furthermore, the rapid epidemiological emergence of MDR species like *C. auris* redefines modern clinical priorities. Fungal vaccine development can no longer rely on narrow, species-specific antigens. Instead, platforms must intentionally evolve toward multi-species, pan-fungal strategies. Achieving this requires global research consortia to overcome the massive financial, regulatory, and logistical hurdles of organizing and funding large-scale Phase III multi-center trials [4,16].

## 4. Reverse Vaccinology 2.0, Transfer Immunotherapy Repertoire, Dual-Action Nanovaccines

The future of immunization against *Candida* species relies on reverse vaccinology 2.0 and structural immunoinformatics [4,7]. This framework uses subtractive proteomics to identify priority target antigens directly from complete digital genomes. This digital screening bypasses the need to culture live fungi in a laboratory, which is time-consuming, expensive, and risky when dealing with a readily transmissible healthcare-associated pathogen capable of causing persistent outbreaks, such as *C. auris* [7]. The design of synthetic, chimeric multi-epitope vaccines allows for the precise selection of peptide fragments with high affinity for human leukocyte antigen (HLA) alleles. Combining fragments from multiple protective proteins, such as Sap2, is predicted to provide global HLA population coverage of over 90% to 94% *in silico* [4,8,9], though this remains a computational estimate not yet validated experimentally. Despite the computational efficiency of reverse vaccinology 2.0 and subtractive proteomics, *in silico* epitope prediction exhibits severe inherent limitations that frequently hinder translational success. High-affinity binding predicted for human leukocyte antigen (HLA) alleles does not systematically translate into robust *in vivo* immunogenicity or protective T-cell responses. Crucially, computational models often fail to account for post-translational modifications (PTMs), particularly the extensive N- and O-linked glycosylation characteristic of the *Candida* cell wall, which can sterically mask predicted peptide epitopes from immune recognition. Furthermore, high mutational plasticity across *Candida* strains risks rapid antigenic drift and epitope escape, rendering static chimeric constructs ineffective against emerging clinical isolates. These computational mismatches explain why numerous high-ranking *in silico* candidates fail during subsequent in vitro functional assays or *in vivo* animal validation. Subunit vaccines of this kind are generally expected to have a favorable safety profile compared with whole-cell or live preparations, given their defined molecular composition, although *in vivo* and clinical safety data for these specific Candida constructs are not yet available. They can be produced rapidly at scale using industrial heterologous expression systems, such as *Escherichia coli* [9]. This scalability paves the way for personalized formulations or vaccines tailored for complex co-infection contexts, a critical need highlighted during the COVID-19 pandemic [7,9].

Beyond nucleic-acid and multi-epitope platforms, the immunotherapy repertoire also extends to *ex vivo* cellular strategies. Dendritic cells loaded *ex vivo* with specific *Candida* antigens have been explored as a way of directly presenting optimized fungal targets to native lymphocytes, bypassing the antigen-presentation bottlenecks that limit conventional vaccination in profoundly immunosuppressed hosts [16]. This cellular approach illustrates that the immunological toolkit available against *Candida* now extends well beyond passive antibody administration or protein/nucleic-acid antigens alone.

This transition toward advanced immunotherapeutic protocols is driven by a deeper understanding of host defense mechanisms. Adjuvant strategies based on recombinant cytokines, such as IFN-γ, IL-7, and GM-CSF, or the adoptive transfer of immune cells expanded *ex vivo*, offer a critical pathway to restore host immunocompetence. These targeted interventions may help counteract immune paralysis in critically ill patients and have been proposed to potentiate the clearance effect of traditional fungicidal drugs. This synergy positions immunotherapy as an increasingly important complementary strategy in an era dominated by MDR strains. Consequently, modern vaccinology and advanced immunotherapies are no longer viewed merely as isolated prophylactic or therapeutic tools. Instead, they function as mechanistic molecular probes that explain the complex, dynamic interaction between the host and the genus *Candida*, revealing novel targets to systematically reverse immune paralysis [17].

**Figure 3:**
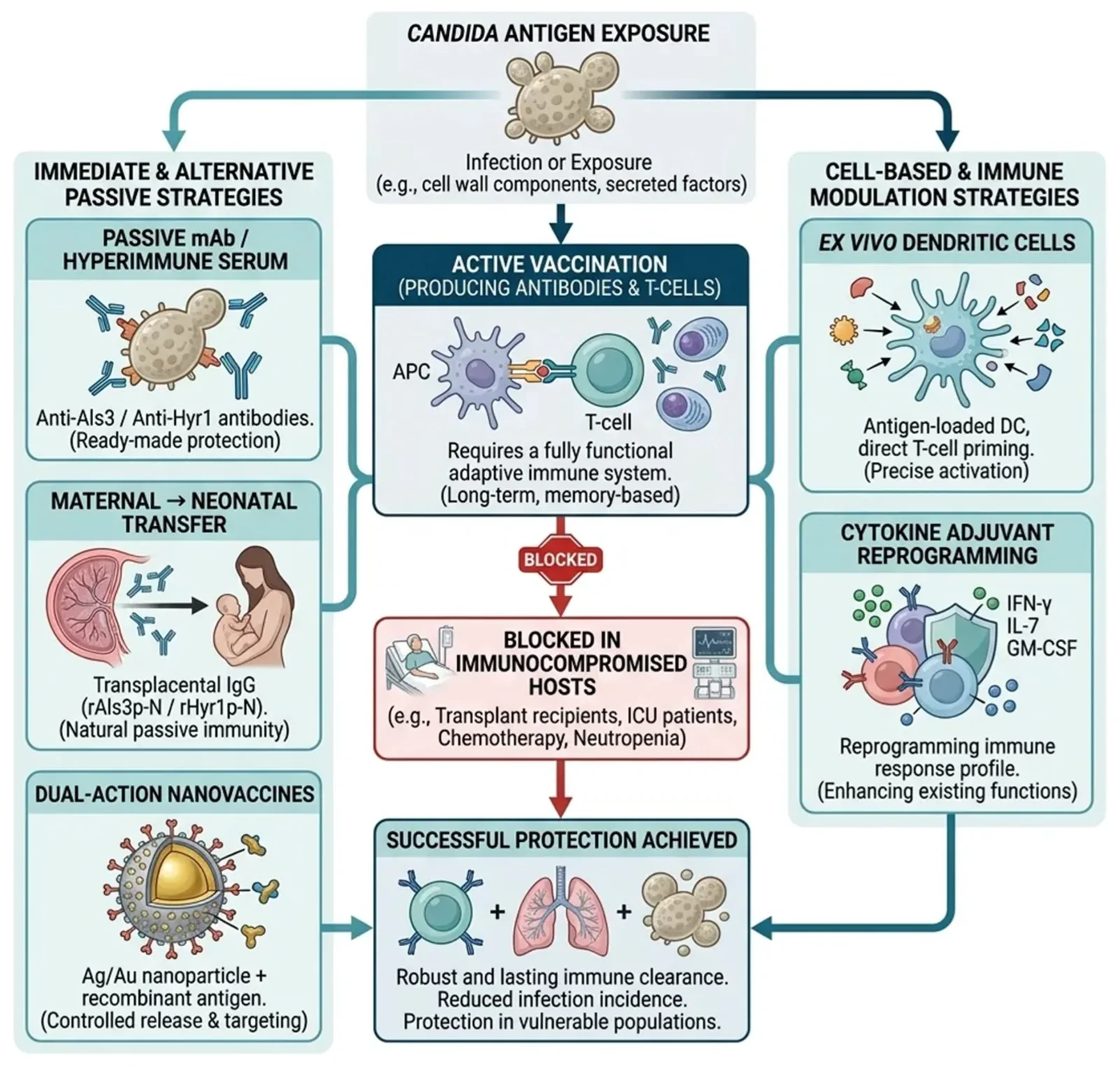
Immunotherapeutic bypass strategies to overcome host immunosuppression, stratified by translational maturity. Conventional active vaccination relies on a functional adaptive immune system, which is blocked in immunocompromised hosts (e.g., transplant recipients, ICU patients, chemotherapy, neutropenia). Alternative strategies circumvent this paradox and are categorized by clinical readiness: Clinical Stage (completed/ongoing Phase I/II evaluation), including recombinant cytokine adjuvant reprogramming (IFN-γ, IL-7, GM-CSF); Advanced Preclinical Stage, featuring passive monoclonal antibodies/hyperimmune sera (e.g., Anti-Als3, Anti-Hyr1) and maternal-to-neonatal transplacental IgG transfer (rAls3p-N/rHyr1p-N); and Early Preclinical Innovations, comprising *ex vivo* antigen-loaded dendritic cell priming and dual-action nanovaccines combining Ag/Au nanoparticles with recombinant antigens. The "Clinical Stage" designation reflects the clinical-trial experience of these recombinant cytokines (IFN-γ, IL-7, GM-CSF) as adjunctive immunotherapy in invasive fungal disease more broadly, rather than an approved or advanced-stage indication for *Candida* vaccine development specifically.

Passive immunization (Figure 3) strategies extend even to the most immunologically vulnerable patients: preterm neonates, whose immature immune systems place them outside the scope of conventional active vaccination. In a neonatal mouse model, maternal immunization with the recombinant antigens rAls3p-N or rHyr1p-N, formulated with Alhydrogel, resulted in transplacental transfer of specific IgG antibodies to offspring, conferring robust protection against a *C. albicans* challenge and significantly reducing renal fungal burden [17,18]. Direct treatment of newborns with hyperimmune serum containing anti-Als3 and anti-Hyr1 antibodies produced comparable protection, including against fluconazole-resistant strains, and cell-depletion experiments confirmed that this antibody-mediated protection strictly depends on functional neutrophils in the neonatal host—mechanistically linking transferred adaptive immunity to the newborn’s own innate defenses [18]. Maternal and neonatal passive immunization therefore represent a promising preclinical strategy for extending protection to a population that active vaccination, by design, cannot reach.

Beyond synthetic chemical compounds, contemporary pharmacology focuses on exploiting natural products and phytotherapeutics as viable alternatives to bypass MDR. Recent data highlight that compounds like berberine, resveratrol, and curcumin act synergistically when co-administered with classical antifungals [19]. Additionally, compounds such as rosmarinic acid and geraniol exhibit a pivotal capacity to inhibit biological virulence factors. By blocking the morphological yeast-to-hypha transition and destabilizing the exopolysaccharide matrix of mature *Candida* biofilms, these molecules render the pathogen significantly more susceptible to host immunological clearance [19].

A promising future direction is the development of dual-action therapies, exemplified by metallic nanovaccines [20]. By combining the prophylactic potential of recombinant surface antigens with the intrinsic antifungal activity of silver and gold nanoparticles, these formulations allow for simultaneous infection prevention and the direct inhibition of fungal growth, while accelerating the regeneration of injured tissues, representing a true fusion between vaccinology and classical pharmacology. While dual-action metallic nanovaccines (e.g., silver or gold nanoparticles conjugated with recombinant antigens) offer potent antimicrobial and adjuvant properties, their path to clinical translation is heavily constrained by long-term toxicological and regulatory bottlenecks. Short-term rodent assays fail to capture the risks of bioaccumulation in parenchymal organs (primarily the liver, spleen, and kidneys), chronic low-grade inflammatory responses, or potential vascular toxicity following systemic administration. From a regulatory perspective (echoing the manufacturing barriers detailed in Section 3), regulatory agencies such as the FDA and EMA demand exhaustive characterization of nanoparticle clearance kinetics, batch-to-batch physicochemical reproducibility, and long-term human safety profiles. These rigorous manufacturing and safety requirements create prolonged approval timelines that significantly disincentivize private biopharmaceutical investment in nanostructure-based antifungal candidates.

The translational readiness of such nanoformulated candidates depends not only on demonstrated efficacy but on demonstrable physicochemical stability. For cationic DODAB: monoolein liposomal platforms, particle size—typically maintained between 100 and 200 nm—and surface charge are decisive for immune-cell uptake; Dynamic Light Scattering and Zeta-potential analyses have confirmed that incorporating fungal cell-wall surface protein extracts does not compromise particle stability [21]. The same lipid platform has since been used to encapsulate the recombinant enzyme Chitinase 3, maintaining a favorable nanometric diameter and a stabilizing cationic Zeta potential [22], and to deliver a synthetic dual-target chimeric peptide (E7–Hyr1), in which lipid encapsulation improved thermodynamic stability and allowed the nanostructured vehicle to trigger stronger dendritic-cell maturation and IL-12/IFN-γ secretion than the free, unencapsulated peptide [23]. These biophysical quality-control steps are not a minor technical footnote: they are precisely the kind of manufacturing evidence regulators require before advanced delivery platforms can move toward clinical testing (see Section 3).

## 5. One Health Approach, Diagnostics, and Global Translational Equity

*Candida auris’* capacity to transmit efficiently within healthcare environments has made it a priority target for hospital infection control on a global scale [1,3,7]. Addressing this threat effectively requires situating vaccine development within a One Health framework: the control of fungal infection can no longer be confined to the hospital, since the interconnection between the environment, agricultural antifungal selection pressure, and human health increasingly shapes which resistant strains reach the clinic in the first place. Vaccination, in this view, could in principle complement environmental and agricultural interventions as part of an integrated One Health strategy against emerging resistant Candida species, although no such vaccination strategy currently exists or has been piloted at the environmental or agricultural level. Furthermore, engaging in an interdisciplinary analysis that compares approaches to vaccine development in animals and humans strengthens the argument for the fundamental nature of existing obstacles in this area, highlighting shared challenges in antigen delivery, safety profiling, and industrial scaling.

Future directions in the fight against the genus *Candida* converge toward a systematic integration of multi-omic technologies, early diagnosis, and a global health perspective. In diagnostics, transitioning from “blind” empirical treatments to early, targeted therapies relies on identifying immunodominant epitopes, such as disaccharide 19, and deploying rapid diagnostic tools like Matrix-Assisted Laser Desorption/Ionization Time-of-Flight (MALDI-TOF) mass spectrometry and real-time PCR [24,25]. The integration of rapid, non-culture-based molecular diagnostic methods and the tight monitoring of specific serum biomarkers stand as crucial pillars of precision medicine. These methods reduce diagnostic delay, allowing earlier targeted therapy and thereby mitigating the high mortality rates associated with delayed diagnosis [11].

This shift toward precision diagnostics is itself grounded in high-throughput molecular characterization. Synthetic glycan microarrays, for example, have mapped the earliest humoral responses to *C. auris* infection, revealing that IgM antibodies specifically target the chemically synthesized β-1,2-mannose disaccharide identified as the minimal immunodominant epitope for this species [25]. Replacing heterogeneous, crude cell-wall extracts with such chemically defined probes provides a level of resolution that could plausibly be translated into rapid, antigen-specific diagnostic assays, complementing MALDI-TOF- and PCR-based approaches. Still, significant challenges persist. The high polymorphism of the Major Histocompatibility Complex (MHC/HLA) in the human population creates wide variations in vaccine efficacy, and marked inequities remain in access to medical innovation across low-income regions where the disease burden is highest [8,24].

Beyond these immunological and economic barriers, translating vaccine candidates from the bench to the global clinical arena faces a severe logistical bottleneck: biological product stability. Unlike traditional chemical antifungals, subunit vaccines, complex glycoconjugates, and live-vector platforms require highly rigorous storage conditions to prevent protein denaturation and the subsequent loss of immunogenic activity [26]. The absolute requirement for a continuous, complex cold chain, paired with the prohibitive costs of large-scale purified protein bio-manufacturing, represents a critical barrier threatening the implementation of these technologies in developing nations.

The scale of this challenge is illustrated by fungal extracellular vesicles (EVs), an acellular vaccine platform increasingly considered for neutropenic patients. Recent characterization of isolated *C. albicans* EVs found that these nanoscale vesicles preserve their membrane integrity and characteristic size distribution even after prolonged storage at 4 °C, −20 °C, and −80 °C [27], directly addressing one of the most critical cold-chain barriers facing biogenic vaccine platforms. Findings of this kind—demonstrating that a candidate tolerates real-world storage conditions rather than only idealized laboratory ones—are precisely what will determine whether next-generation *Candida* vaccines can reach low-resource settings, rather than remaining confined to well-resourced healthcare systems.

## 6. Conclusions

The absence of a licensed *Candida* vaccine, more than a decade after the first adhesin-based candidates entered clinical testing, cannot be attributed to a single failure point. The barriers are layered: biological, in a pathogen whose commensal identity and morphological plasticity actively resist a one-size-fits-all immune target; technical, in preclinical models that have only recently begun to replicate genuine host susceptibility rather than chemically imposed immunosuppression; and structural, in a regulatory and commercial environment that treats fragmented, high-risk patient populations as poor investment targets rather than as the populations most in need of protection.

Emerging immunotherapeutic strategies—passive monoclonal antibodies, cytokine-based adjuvant reprogramming, phytocompound-derived antifungals, and dual-action nanovaccines—represent meaningful progress, but they are best understood as ways of working around the immunocompromised-host paradox rather than resolving it. A vaccine that requires a functional adaptive immune system will always struggle in the transplant recipients, ICU patients, and chemotherapy patients who make up much of the target population; passive and cell-based approaches partially close that gap without eliminating the underlying tension.

Finally, none of these scientific advances will translate into global protection without deliberate attention to diagnostic infrastructure, HLA population diversity, and vaccine storage economics—factors that determine whether a licensed product would actually reach low-resource settings where the burden of candidiasis, and of antifungal resistance, is often highest. We suggest that future *Candida* vaccine research explicitly integrate these One Health and equity considerations from early development, rather than treating them as implementation details to be solved after licensure. Closing the translational gap in antifungal vaccinology will depend as much on addressing these structural and global barriers as on further antigen discovery.

## Figures and Tables

**Table 1:** Shifting Epidemiology of Fungal Pathogens.

Fungal Pathogen	Clinical Impact & Challenges	Hospital Mortality Rate
*C. albicans*	Historically dominant species Transitions easily between yeast and hyphae.	Baseline Risk
*C. glabrata*	Rising incidence Displays intrinsic (reduced-susceptibility) resistance to azoles; acquired echinocandin resistance can also emerge, particularly under prior echinocandin exposure.	30–50%
*C. parapsilosis*	Rising incidence Reduced intrinsic susceptibility to echinocandins (naturally occurring *FKS1* hotspot polymorphism), with emerging acquired azole resistance in some clinical isolates.	30–50%
*C. auris*	Emerging global threat. Forms resilient biofilms and resists multiple drug classes.	30–50%

**Table 2:** *C. auris* clearance pathway insight.

Model	Outcome
Neutrophil Elastase Deficient Mice	Remain resistant to *C. auris*
C5 Complement Deficient A/J Mice	Suffer fulminant, lethal infection
Interpretation	Resistance to *C. auris* depends critically on the complement cascade, not solely on isolated neutrophil enzymatic activity

**Table 3:** The regulatory and formulation hurdles.

Barrier	Description
Patient Cohort Split	Fragmented: high-risk groups vs universal cohorts
Target Delivery Systems	Nanoparticles & hydrogels lack long-term human safety records
Biophysical Profile	Inherent pharmacokinetic and storage instability risks

**Table 4:** The antifungal funding gap.

	Universal Viral/Bacterial Vaccines	*Candida* Target Vaccines
Target	Global population	Fragmented niches
Market Size	Billions	High-risk only
Return on Investment	High	Low
Priority status	High priority	Chronic shortage

**Table 5:** Technical horizons in clinical platforms.

Platform	Characteristics
Recombinant Proteins	Highly purified targets, require potent adjuvants
Virosomes (PEV7)	Local mucosal delivery, targets specific portals of entry
Glucan Particles	Fungal cell-wall mimics, intrinsic self-adjuvating assets

## Data Availability

The data is available upon request to the corresponding author.
